# Purinergic signaling promotes premature senescence

**DOI:** 10.1016/j.jbc.2024.107145

**Published:** 2024-03-07

**Authors:** Daniela Volonte, Cory J. Benson, Stephanie L. Daugherty, Jonathan M. Beckel, Mohamed Trebak, Ferruccio Galbiati

**Affiliations:** 1 Department of Pharmacology & Chemical Biology, University of Pittsburgh School of Medicine, Pittsburgh, Pennsylvania, USA; 2 Vascular Medicine Institute, University of Pittsburgh School of Medicine, Pittsburgh, Pennsylvania, USA

**Keywords:** senescence, ATP, purinergic signaling, reactive oxygen species, triple negative breast cancer cells

## Abstract

Extracellular ATP activates P2 purinergic receptors. Whether purinergic signaling is functionally coupled to cellular senescence is largely unknown. We find that oxidative stress induced release of ATP and caused senescence in human lung fibroblasts. Inhibition of P2 receptors limited oxidative stress-induced senescence, while stimulation with exogenous ATP promoted premature senescence. Pharmacological inhibition of P2Y11 receptor (P2Y11R) inhibited premature senescence induced by either oxidative stress or ATP, while stimulation with a P2Y11R agonist was sufficient to induce cellular senescence. Our data show that both extracellular ATP and a P2Y11R agonist induced calcium (Ca^++^) release from the endoplasmic reticulum (ER) and that either inhibition of phospholipase C or intracellular Ca^++^ chelation impaired ATP-induced senescence. We also find that Ca^++^ that was released from the ER, following ATP-mediated activation of phospholipase C, entered mitochondria in a manner dependent on P2Y11R activation. Once in mitochondria, excessive Ca^++^ promoted the production of reactive oxygen species in a P2Y11R-dependent fashion, which drove development of premature senescence of lung fibroblasts. Finally, we show that conditioned medium derived from senescent lung fibroblasts, which were induced to senesce through the activation of ATP/P2Y11R-mediated signaling, promoted the proliferation of triple-negative breast cancer cells and their tumorigenic potential by secreting amphiregulin. Our study identifies the existence of a novel purinergic signaling pathway that links extracellular ATP to the development of a protumorigenic premature senescent phenotype in lung fibroblasts that is dependent on P2Y11R activation and ER-to-mitochondria calcium signaling.

Cellular senescence is a stable form of cell cycle arrest (1, 2, 3, 4). Replicative senescence is dependent on the number of divisions the cell has completed. Senescence can be accelerated by several stressful stimuli, including oxidative stress and UV light exposure (5, 6, 7, 8, 9). This type of senescence is referred to as stress-induced premature senescence. Senescent cells are characterized by an enlarged and flat morphology and well-defined molecular changes, including increased β-galactosidase activity at pH 6, p16 and p21^Waf1/Cip1^ protein expression, and enhanced p53 activity (5, 6, 7, 8, 9, 10, 11). Senescent cells are also characterized by a chronic DNA damage response with elevated γ-H2A.X expression, formation of DNA damage (10), and senescence-associated heterochromatin (11) foci. Moreover, senescent cells secrete a plethora of cytokines, growth factors and proteases, known as the senescence-associated secretory phenotype (12, 13, 14, 15, 16, 17, 18, 19, 20). Senescent cells accumulate in tissues over time and contribute to both aging and the development of age-associated diseases (21, 22, 23, 24, 25, 26, 27). Senescent cells have antagonistic pleiotropic roles in cancer. Since senescent cells are unable to proliferate, cellular senescence is a powerful tumor suppressor mechanism (28, 29, 30, 31, 32, 33). However, accumulation of senescent cells during aging can promote tumor growth given their ability to release factors that stimulate cancer cell proliferation (18, 19, 20, 34, 35). The signaling pathways and molecular mechanisms that regulate the acquisition of a senescent phenotype are therefore of upmost importance in both aging and cancer biology.

ATP provides energy to drive a plethora of cellular processes. In the seventies, ATP was proposed to be a signaling molecule both in the peripheral and central nervous systems (36). Since then, evidence has shown that ATP is a critical extracellular signal transduction molecule also in other tissues and in multiple cell types (36, 37, 38, 39). Intracellular ATP is released into the extracellular space in response to a variety of stimuli, such as oxidative stress, shear stress, ionizing radiation, stretch, and hypoxia (37, 40, 41). Cells release ATP through different mechanisms (37, 42, 43). Among them, connexin (Cx) hemichannels (44, 45, 46) and pannexin (Panx) channels (47, 48, 49, 50) are key mediators of ATP release. Extracellular ATP activates P2 purinergic receptors. P2 receptors have been subclassified as either metabotropic P2Y receptors or ionotropic P2X receptors (51). Multiple subtypes exist of both the P2Y and P2X receptors, which are differentially expressed in many organs and tissues (52, 53). P2Y receptors are G-protein coupled receptors that couple mostly to G_q/11_, but also G_i/o_ and G_s_, leading to changes of intracellular levels of Ca^++^ and cAMP (54, 55, 56). P2X receptors are ligand-gated ion channels that allow the flux of mostly Ca^++^, Na^+^, and K^+^ ions (57, 58, 59). If autocrine purinergic signaling is functionally coupled to cellular senescence is largely unknown.

In the present study, we find that P2 purinergic signaling is causally coupled to the development of premature senescence. We provide evidence that a new signaling pathway exists in human lung fibroblasts that is initiated by extracellular ATP and is transduced by the P2Y11R/phospholipase C (PLC)-mediated release of calcium from intracellular stores, which then enters mitochondria and promotes free radical production and reactive oxygen species (ROS)-dependent and p53/p21-mediated cellular senescence. We also find that ATP-mediated senescence of lung fibroblasts promotes the growth and enhances the tumorigenic potential of triple-negative breast cancer (TNBC) cells through the release of amphiregulin. Together, these findings provide novel insights into the molecular mechanisms that control the development of a premature senescent phenotype, a cellular event that is relevant to the fields of aging, age-related disease, and cancer.

## Results

### Extracellular ATP signaling mediates stress-induced premature senescence through P2 purinergic receptor activation

**Figure 1:**
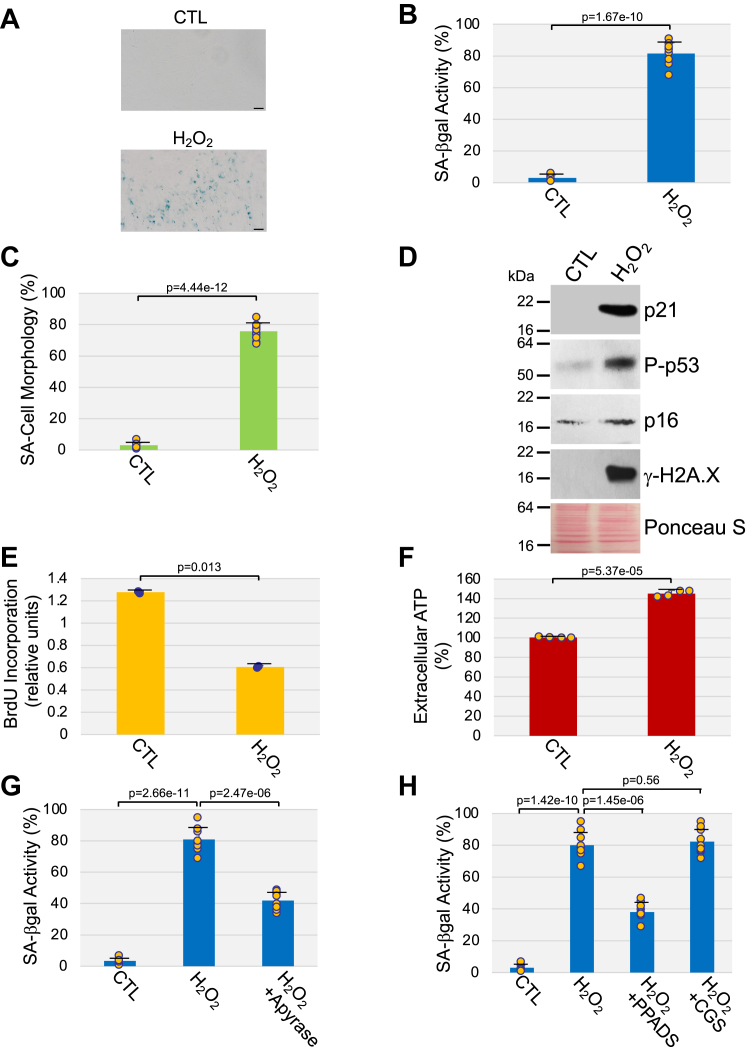
**Release of extracellular ATP mediates oxidative stress-induced premature senescence in human fibroblasts through P2 receptor activation.** WI-38 human diploid fibroblasts were treated with sublethal hydrogen peroxide (450 μM H_2_O_2_ for 2 h). Cells were washed with PBS and recovered in complete medium for 10 days. Untreated cells were used as control. *A* and *B,* cells were stained to detect senescence-associated β-galactosidase activity. Representative images are shown in (*A*), quantification is shown in (*B*). The percentage of cells possessing enlarged and flat morphology [senescence-associated (SA) cell morphology] is shown in (*C*). *D*, cells were collected, and cell lysates were subjected to immunoblot analysis using protein-specific antibody probes. Ponceau S staining shows equal total protein loading. *E*, cell proliferation was quantified by BrdU incorporation assay. *F*, the level of extracellular ATP was quantified in the conditioned medium using an ATP bioluminescent assay kit. *G* and *H*, cells were treated with 450 μM H_2_O_2_ for 2 h and recovered in complete medium for 10 days in the presence of either 4 U/ml apyrase (*G*), 500 μM PPADS (*H*), or 5 μM CGS 15943 (*H*). Untreated cells served as control. Quantification of senescence-associated β-galactosidase activity is shown. Values in *B*, *C*, and (*E*–*H*) represent means ± SD; statistical comparisons were made using the student’s *t* test. The scale bar represents 50 μm. BrdU, bromodeoxyuridine; CGS 15943, 9-chloro-2-(2-furanyl)-[1,2,4]triazolo[1,5-*c*]quinazolin-5-amine; H_2_O_2_, hydrogen peroxide; PPADS, pyridoxal-phosphate-6-azophenyl-2′,4′-disulfonic acid tetrasodium salt.

The role that purinergic signaling plays in cellular senescence is largely unknown. To begin investigating the role of ATP in stress-induced premature senescence, we asked whether cells that are hit by stressors known to induce premature senescence release extracellular ATP. To this end, we exposed WI-38 human diploid lung fibroblasts to oxidative stress by treating the cells with sublethal levels of hydrogen peroxide (H_2_O_2_). Sublethal oxidative stress induced senescence in WI-38 cells, as shown by quantification of cells that are positive for senescence-associated β-galactosidase (SA-β-gal) activity (Fig. 1, *A* and *B*) and senescence-associated cell morphology (Fig. 1*C*), by immunoblotting analysis using antibodies specific for the senescence markers phospho-p53, p21, p16, and γ-H2A.X (Fig. 1*D*), and by bromodeoxyuridine (BrdU) incorporation assay (Fig. 1*E*).

We find that the level of extracellular ATP was significantly increased in senescent WI-38 cells following oxidative stress stimulation (Fig. 1*F*). Interestingly, treatment with apyrase, an enzyme that catalyzes the hydrolysis of ATP to AMP, inhibited the development of oxidative stress-induced premature senescence in these cells (Figs. 1*G* and S1*A*). We also find that treatment with the nonselective P2 purinergic receptor antagonist pyridoxal-phosphate-6-azophenyl-2′,4′-disulfonic acid tetrasodium salt (PPADS), but not the adenosine P1 receptor antagonist 9-chloro-2-(2-furanyl)-[1,2,4]triazolo[1,5-*c*]quinazolin-5-amine (CGS 15943), inhibited premature senescence induced by oxidative stress in WI-38 fibroblasts (Figs. 1*H* and S1*B*). Consistent with these data, we find that treatment with ultraviolet C (UV-C) light also induced premature senescence (Fig. S1, *C*–*E*) and promoted the release of ATP (Fig. S1*F*).

Thus, ATP is released by human fibroblasts following exogenous stress and contributes to the development of a premature senescent phenotype through the activation of P2 purinergic receptors.

### Stimulation of P2 receptor signaling is sufficient to induce premature senescence

**Figure 2:**
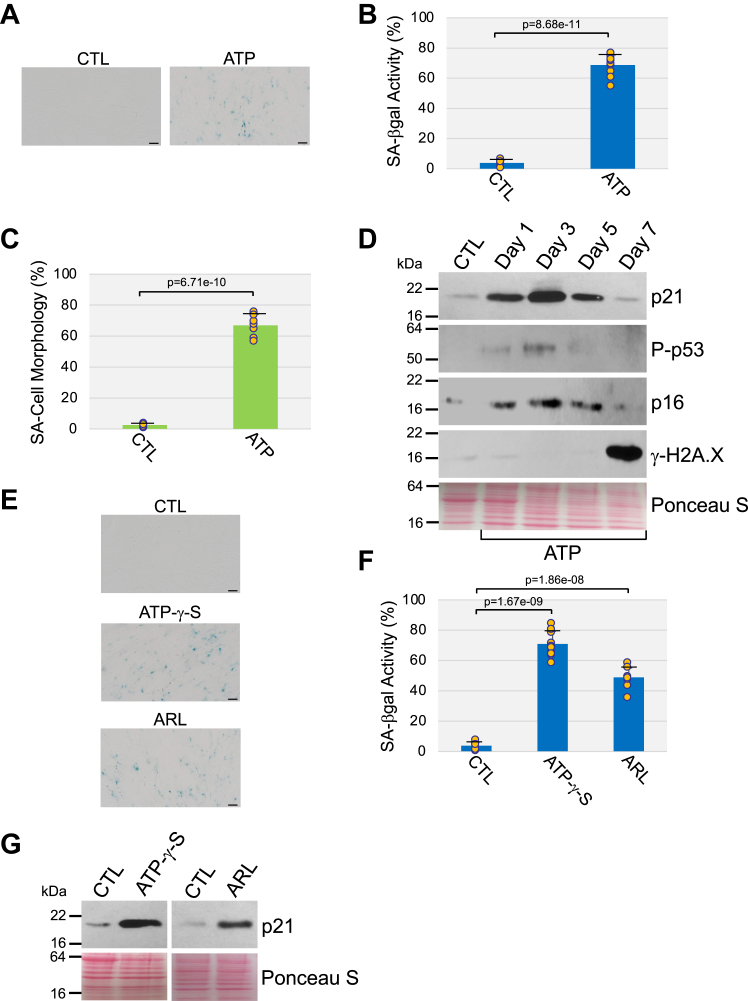
**Stimulation with ATP is sufficient to promote premature senescence in human fibroblasts.** Human diploid WI-38 fibroblasts were treated with 1.5 mM ATP for 10 days. Untreated cells were used as control. *A* and *B*, cells were subjected to senescence-associated β-galactosidase activity staining. Representative images are shown in (*A*), quantification is shown in (*B*). The percentage of cells possessing enlarged and flat morphology [senescence-associated (SA) cell morphology] is shown in (*C*). *D*, cells were collected, and cell lysates were subjected to immunoblot analysis using antibody probes specific for p21, phospho-p53, p16, and γ-H2A.X. Ponceau S staining shows equal total protein loading. *E*–*G*, WI-38 cells were treated with either 400 μM ATP-γ-S or 400 μM ARL 67156 for 10 days. Untreated cells served as control. *E* and *F*, cells were stained to detect senescence-associated β-galactosidase activity. Representative images are shown in (*E*), quantification is shown in (*F*). *G*, the expression level of the senescence marker p21 was quantified by immunoblotting analysis using a p21-specific antibody probe. Ponceau S staining shows equal total protein loading. Values in *B*, *C*, and *F* represent means ± SD; statistical comparisons were made using the student’s *t* test. The scale bar represents 50 μm.

To determine if ATP stimulation can induce cellular senescence in the absence of an external stressor, we treated WI-38 fibroblasts with ATP for 10 days. Our data show that ATP stimulation was sufficient to induce premature senescence, as demonstrated by the accumulation of cells that were positive for SA-β-gal activity (Fig. 2, *A* and *B*) and that displayed senescence-associated cell morphology (Fig. 2*C*), and by the upregulation of the senescence markers P-p53, p21, p16, and γ-H2A.X (Fig. 2*D*). It is worthy of note that ATP concentrations ranging from 10 μM to 1.5 mM induced senescence in WI-38 cells without causing any cell death (not shown). Since 1.5 mM ATP induced the highest degree of senescence, we chose to use 1.5 mM ATP stimulation for 10 days in all subsequent experiments. ATP is hydrolyzed to ADP and AMP by CD39, and to adenosine (ADO) by CD73 ectonucleotidases. We find that either ATPγS, a nonhydrolyzable form of ATP, or ARL 67156, an inhibitor of ATP breakdown, promoted senescence in human fibroblasts (Fig. 2, *E*–*G*). In support of these data, apyrase inhibited ATP-induced senescence (Fig. S2, *A*–*C*). We conclude that ATP itself, and not ATP metabolites, promotes premature senescence in human fibroblasts.

**Figure 3:**
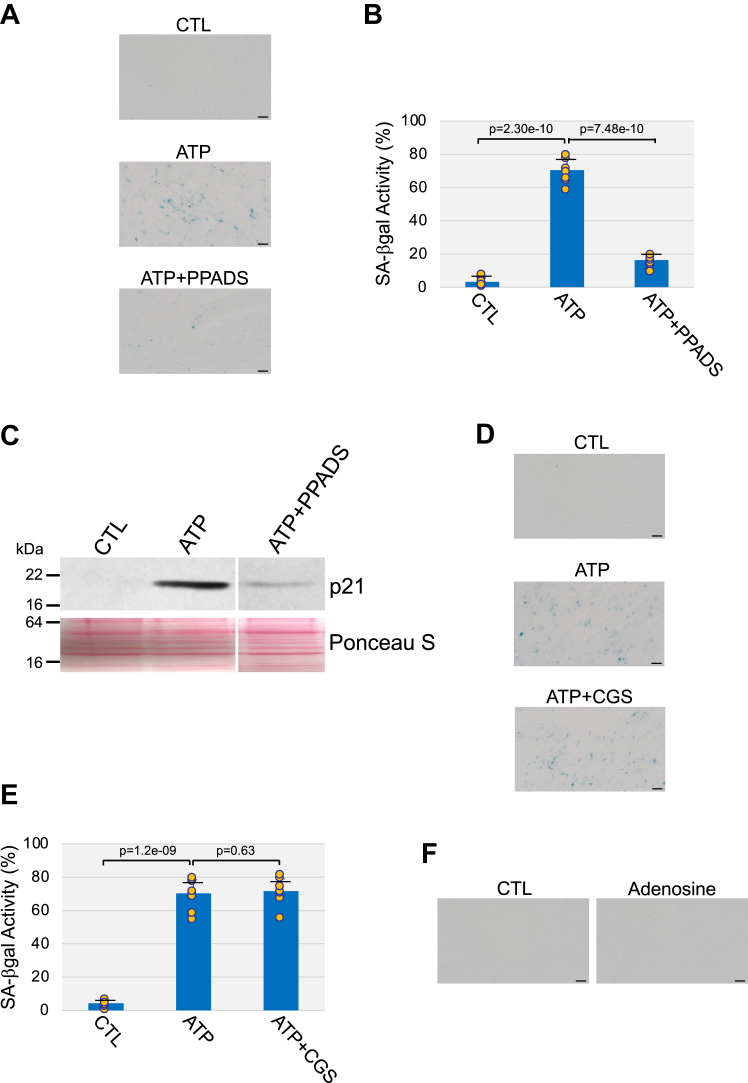
**Activation of P2 receptors mediates premature senescence induced by ATP.***A*–*C*, WI-38 cells were treated with 1.5 mM ATP for 10 days in the presence or absence of the nonspecific P2R antagonist PPADS (500 μM). Untreated cells were used as control. Cells were stained to detect senescence-associated β-galactosidase activity. Representative images are shown in (*A*), quantification is shown in (*B*). *C*, cells were collected and cell lysates were subjected to immunoblot analysis using an antibody probe specific for the senescence marker p21. Ponceau S staining shows equal total protein loading. *D* and *E*, WI-38 human diploid fibroblasts were treated with 1.5 mM ATP for 10 days in the presence or absence of 5 μM CGS 15943. Untreated cells served as control. Cells were subjected to senescence-associated β-galactosidase activity staining. Representative images are shown in (*D*), quantification is shown in (*E*). *F*, WI-38 fibroblasts were treated with 1.5 mM adenosine for 10 days. Untreated cells served as control. Senescence was quantified by senescence-associated β-galactosidase activity staining. Representative images are shown. Values in *B* and *E* represent means ± SD; statistical comparisons were made using the student’s *t* test. The scale bar represents 50 μm. CGS 15943, 9-chloro-2-(2-furanyl)-[1,2,4]triazolo[1,5-*c*]quinazolin-5-amine; PPADS, pyridoxal-phosphate-6-azophenyl-2′,4′-disulfonic acid tetrasodium salt.

Our data also show that ATP induced senescence by activating P2 purinergic receptors, as demonstrated by the ability of PPADS to inhibit ATP-induced senescence in WI-38 cells (Fig. 3, *A*–*C*). In contrast, inhibition of P1 purinergic receptors with CGS 15943 failed to inhibit senescence induced by ATP (Fig. 3, *D* and *E*). In addition, treatment with adenosine for 10 days did not induce senescence in WI-38 fibroblasts (Figs. 3*F* and S2*D*). Thus, our findings show that ATP promotes premature senescence by activating a P2 receptor-mediated intracellular signaling.

### P2Y11 receptor mediates ATP-induced premature senescence

**Figure 4:**
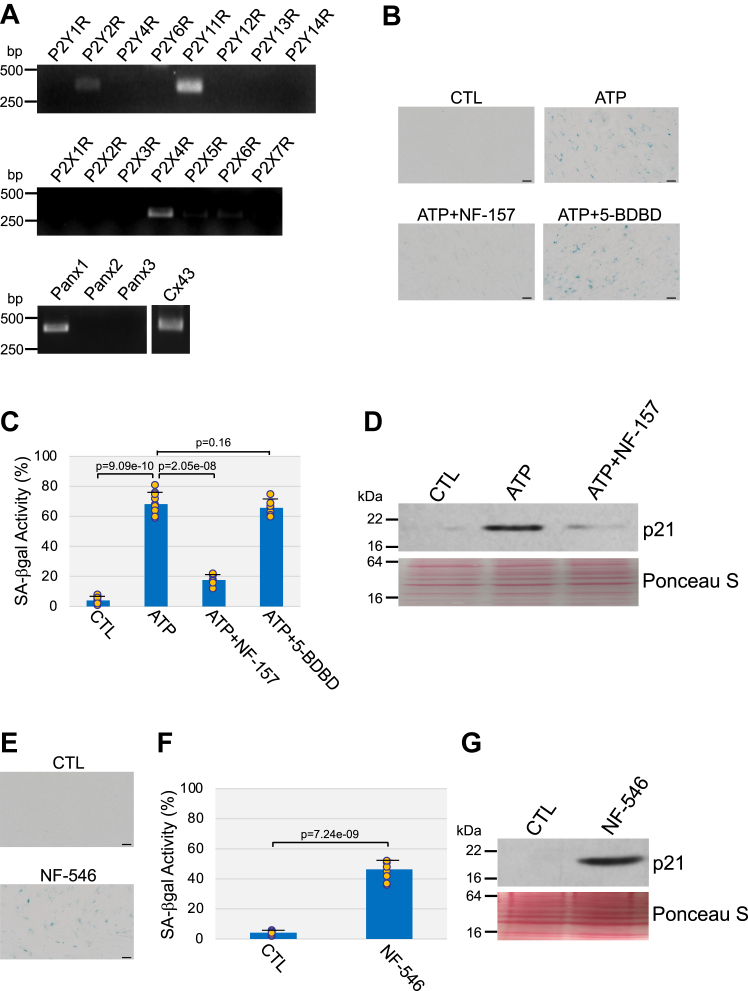
**P2Y11 receptor mediates ATP-induced premature senescence in human fibroblasts.***A*, WI-38 fibroblasts were treated with H_2_O_2_ (450 μM) for 2 h and recovered in complete medium for 10 days to induce senescence. The expression level of P2Y receptors (P2YR), P2X receptors (P2XR), pannexin channels (Panx), and connexin 43 (Cx43) was determined by RT-PCR analysis using mRNA-specific primers. *B* and *C*, WI-38 fibroblasts were treated with 1.5 mM ATP for 10 days in the presence or absence of either 40 μM NF-157 or 40 μM 5-BDBD. Untreated cells were used as control. Cells were stained to detect senescence-associated β-galactosidase activity. Representative images are shown in (*B*), quantification is shown in (*C*). *D*, WI-38 fibroblasts were treated with 1.5 mM ATP in the presence or absence of 40 μM NF-157. Untreated cells served as control. The expression level of the senescence marker p21 was detected by immunoblotting analysis. Ponceau S staining shows equal total protein loading. *E*–*G*, WI-38 fibroblasts were treated with 80 μM NF-546 for 10 days. Untreated cells were used as control. *E* and *F*, cells were stained to detect senescence-associated β-galactosidase activity. Representative images are shown in (*E*), quantification is shown in (*F*). *G*, cells were collected, and cell lysates were subjected to immunoblot analysis using an antibody probe specific for the senescence marker p21. Ponceau S staining shows equal total protein loading. Values in *C* and *F* represent means ± SD; statistical comparisons were made using the student’s *t* test. The scale bar represents 50 μm. H_2_O_2_, hydrogen peroxide.

In order to identify the P2 receptor subtype that mediates the development of cellular senescence, we first determined the mRNA expression profile of P2 purinergic receptors in WI-38 fibroblasts that were induced to senesce by oxidative stress. Our data show that P2Y11 and P2X4 were the main P2 receptor subtypes expressed in senescent human diploid fibroblasts (Fig. 4*A*). Interestingly, WI-38 fibroblasts also expressed pannexin 1 (Panx1) and connexin-43 (Cx43) (Fig. 4*A*), which are known to mediate ATP release from cells. We then induced senescence by treating WI-38 cells with either ATP or ARL 67156 for 10 days in the presence or absence of either NF-157, a P2Y11 receptor-specific antagonist, or 5-BDBD, a selective P2X4 receptor antagonist. We find that inhibition of P2Y11, but not P2X4, receptor inhibited both ATP-induced (Fig. 4, *B*–*D*) and ARL 67156-induced (Fig. S3, *A* and *B*) senescence. Moreover, ATP stimulation inhibited BrdU incorporation in WI-38 cells, which was prevented by NF-157 (Fig. S3*C*). Consistent with these data, NF-157 inhibited oxidative stress-induced premature senescence of WI-38 cells (Fig. S3, *D* and *E*). Importantly, stimulation of WI-38 fibroblasts with NF-546, a selective P2Y11 receptor agonist, was sufficient to induce premature senescence (Figs. 4, *E*–*G* and S3*F*). Together, these data indicate that activation of P2Y11 receptor-mediated signaling promotes premature senescence in human diploid fibroblasts.

### Activation of G_q_-PLC-Ca^++^ signaling mediates premature senescence induced by ATP

**Figure 5:**
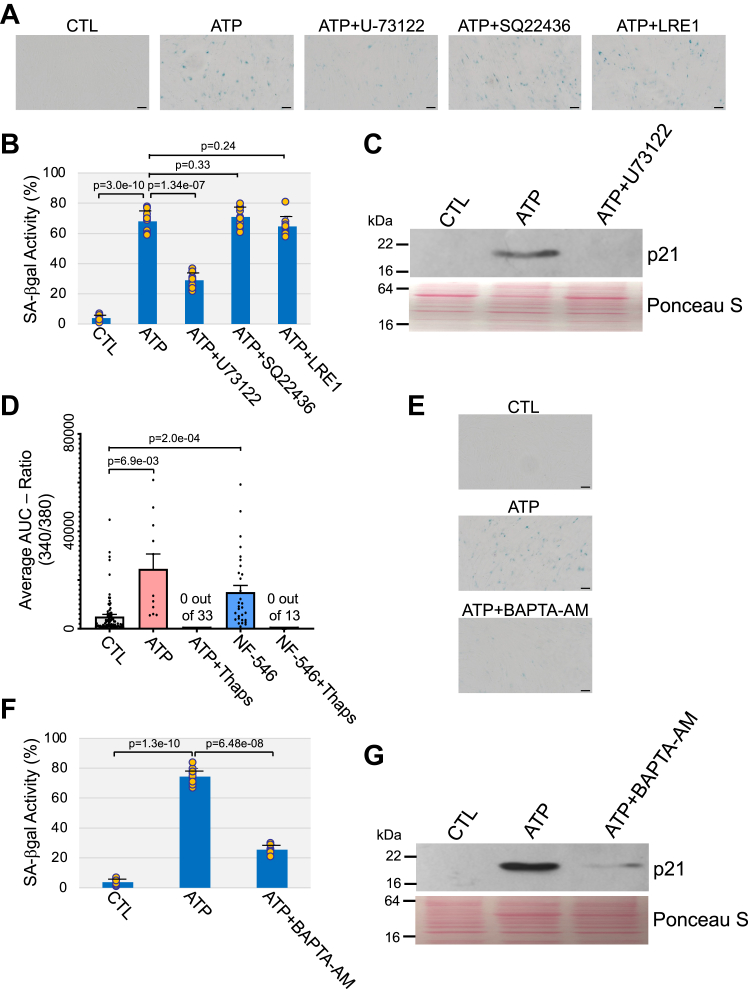
**Inhibition of PLC-mediated calcium release from intracellular stores impairs ATP-induced premature senescence.***A*–*C*, WI-38 human diploid fibroblasts were treated with 1.5 mM ATP for 10 days in the presence or absence of either U-73122 (3 μM), SQ22436 (45 μM), or LRE1 (9 μM). Untreated cells were used as control. *A* and *B*, cells were stained to detect senescence-associated β-galactosidase activity. Representative images are shown in (*A*), quantification is shown in (*B*). *C*, cell lysates were subjected to immunoblotting analysis with anti-p21 IgGs. Ponceau S staining shows equal total protein loading. *D*, intracellular calcium was quantified in WI-38 fibroblasts loaded with Fura-2 AM, before and after either ATP (100 μM) or NF-546 (80 μM) stimulation. Intracellular calcium was also quantified after intracellular stores were preemptively depleted with 1 μM thapsigargin (Thaps) treatment before agonist stimulation. *E*–*G*, WI-38 fibroblasts were treated with 1.5 mM ATP for 10 days in the presence or absence of 5 μM BAPTA-AM. Untreated cells served as control. Cells were subjected to senescence-associated β-galactosidase activity staining. Representative images are shown in (*E*), quantification is shown in (*F*). *G*, cells were also collected and cell lysates were subjected to immunoblot analysis using an antibody probe specific for p21. Ponceau S staining shows equal total protein loading. Values in *B*, *D*, and *F* represent means ± SD; statistical comparisons were made using the student’s *t* test. The scale bar represents 50 μm. PLC, phospholipase C.

The P2Y11 receptor has been shown to couple to mostly G_q_ but also G_s_ and G_i/o_. To identify the signaling mechanism underlying ATP-induced and P2Y11 receptor-mediated premature senescence, we investigated the functional consequence of inhibiting either PLC or adenylyl cyclase (AC) on premature senescence following chronic ATP stimulation for 10 days. We find that inhibition of PLC function with the specific inhibitor U-73122 impaired the upregulation of the senescence markers senescence-associated β-galactosidase activity and p21 induced by ATP in WI-38 fibroblasts (Fig. 5, *A*–*C*). In contrast, inhibition of either membrane AC with SQ 22536 or soluble AC with LRE-1 failed to prevent ATP-induced senescence (Fig. 5, *A* and *B*), suggesting a key role of PLC in ATP/P2Y11 receptor-induced senescence.

Activation of PLC leads to inositol-1,4,5-trisphosphate (IP_3_)-mediated calcium release from the endoplasmic reticulum (ER) into the cytoplasm. We show in Figure 5*D* that treatment with either ATP or the P2Y11 receptor agonist NF-546 elicited a rise of intracellular calcium level in WI-38 cells, which was prevented when intracellular calcium stores were preemptively depleted with thapsigargin treatment. Consistent with these findings, intracellular calcium chelation with BAPTA-AM inhibited ATP-induced premature senescence in WI-38 cells (Fig. 5, *E*–*G*). Thus, release of intracellular calcium from the ER, following ATP-initiated and P2Y11 receptor/PLC-mediated signaling drives premature senescence in human fibroblasts. Interestingly, we did not find increased intracellular calcium levels in ATP-treated WI-38 cells 10 days after ATP treatment that is after the cells have reached a senescent phenotype (Fig. S4), suggesting that ATP-induced release of calcium from the ER plays a key role in promoting senescence but it is not required for the maintenance of a senescent phenotype.

### Calcium uptake into mitochondria promotes ROS production and premature senescence following ATP-initiated and P2Y11 receptor/PLC-mediated calcium release from the ER

How can we explain the functional link between ATP-induced calcium release from the ER into the cytoplasm and induction of premature senescence? Calcium uptake by the mitochondria helps buffer cytosolic calcium transients and couples surface receptor stimulation to mitochondrial metabolism. However, excessive calcium accumulation in mitochondria can lead to ROS generation. Studies show that exposure of isolated mitochondria to calcium increases free radical production (60). Also, treatment of cultured cells with a calcium ionophore promotes mitochondrial ROS generation (61, 62). Mitochondrial Ca^++^-induced ROS production occurs through different mechanisms, including the ability of calcium to stimulate enzymes of the Krebs cycle, leading to increased respiratory chain electron leakage and free radical levels (63). Since oxidative stress can induce premature senescence, we asked whether the ATP/P2Y11R-induced calcium release from the ER into the cytoplasm leads to calcium uptake into mitochondria, ROS production, and ROS-dependent premature senescence.

**Figure 6:**
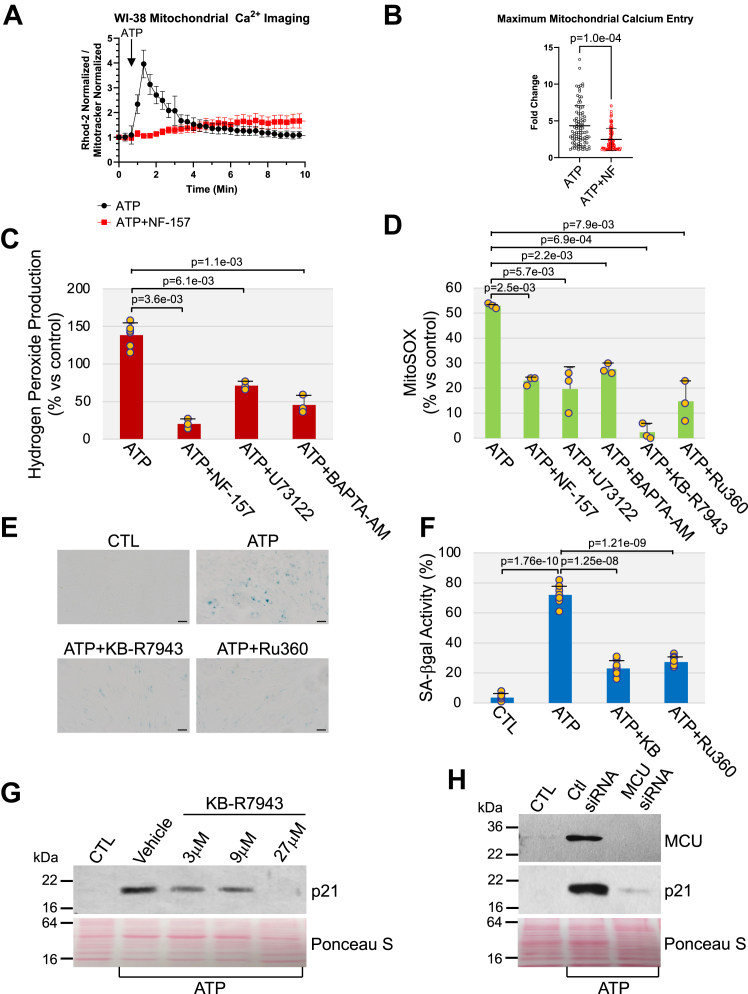
**ATP stimulation promotes calcium accumulation and ROS generation in mitochondria in a P2Y11R-dependent manner.** Inhibition of mitochondrial calcium accumulation impairs ATP-induced premature senescence. *A*, WI-38 fibroblasts were cultured overnight in the presence or absence of NF-157 (40 μM). Cells were then stimulated with ATP (100 μM) and mitochondrial Ca^++^ was measured by dividing the fluorescence of Rhod-2 by MitoTracker Green. *B*, Quantification of mitochondrial calcium uptake from (*A*). *C*, WI-38 cells were treated with 1.5 mM ATP for 10 days in the presence or absence of either 40 μM NF-157, 3 μM U-73122, or 5 μM BAPTA-AM. Untreated cells were used as control. The level of intracellular hydrogen peroxide was quantified by Amplex Red staining. *D*, WI-38 fibroblasts were stimulated with ATP (1.5 mM) for 10 days in the presence or absence of either NF- 157 (40 μM), U-73122 (3 μM), BAPTA-AM (5 μM), KB-R7943 (9 μM), or Ru-360 (9 μM). Untreated cells served as control. The level of mitochondrial superoxide was quantified using the MitoSOX Red superoxide indicator. *E* and *F*, human diploid WI-38 fibroblasts were treated with 1.5 mM ATP for 10 days in the presence or absence of either KB-R7943 (9 μM) or Ru-360 (9 μM). Untreated cells were used as control. Cells were subjected to senescence-associated β-galactosidase activity staining. Representative images are shown in (*E*), quantification is shown in (*F*). *G*, WI-38 cells were treated with 1.5 mM ATP for 10 days in the presence or absence of increasing concentrations of KB-R7943 (3, 9, and 27 μM). Untreated cells were used as control. Cells were collected and cell lysates were subjected to immunoblotting analysis using an antibody probe specific for p21. Ponceau S staining shows equal total protein loading. *H*, WI-38 fibroblasts were transfected with either control (Ctl) siRNA or MCU siRNA. After 24 h, cells were treated with ATP (1.5 mM) for 5 days. Untreated cells served as control. The expression level of both MCU and p21 was detected by immunoblotting analysis using specific antibody probes. Ponceau S staining shows equal total protein loading. Values in *B*, *C*, *D*, and *F* represent means ± SD; statistical comparisons were made using the student’s *t* test. The scale bar represents 50 μm. MCU, mitochondrial calcium uniporter; ROS, reactive oxygen species.

We first show that ATP stimulation induced an increase of calcium in the mitochondrial matrix, which is representative of mitochondrial calcium uptake (Fig. 6, *A* and *B*). ATP-induced calcium uptake in mitochondria was impaired by P2Y11 receptor inhibition (Fig. 6, *A* and *B*). We then show in Figure 6*C* that ATP promoted ROS generation in WI-38 fibroblasts, as assessed by Amplex Red staining, which was prevented if WI-38 cells were cotreated with ATP and either the P2Y11 receptor inhibitor NF-157, the PLC inhibitor U-73122, or the calcium chelator BAPTA-AM. We find that mitochondria are the source of ATP-induced ROS generation, as shown by MitoSox Red staining (Fig. 6*D*). Production of ROS in the mitochondria after ATP stimulation was inhibited when the cells were cotreated with either NF-157, U-73122, or BAPTA-AM (Fig. 6*D*).

The mitochondrial calcium uniporter (MCU) promotes calcium entry in the mitochondria. We find that treatment with the MCU inhibitors KB R7943 mesylate or Ru360 prevented both ROS production in mitochondria (Fig. 6*D*) and upregulation of the senescence markers SA-β-gal activity (Fig. 6, *E* and *F*) and p21 induced by ATP stimulation (Figs. 6*G* and S5*A*). Consistent with these findings, downregulation of MCU by siRNA inhibited ATP-induced upregulation of p21 (Fig. 6*H*). Importantly, treatment of WI-38 fibroblasts with the antioxidant quercetin inhibited premature senescence induced by ATP (Fig. S5, *B*–*D*). We conclude that calcium overload in the mitochondria, which follows ATP-induced activation of P2Y11R and PLC-mediated calcium release from the ER, promotes mitochondrial ROS generation and premature senescence. Our study shows that ER-to-mitochondria signaling plays a key role in premature senescence in response to activation of the P2Y11 purinergic receptor following extracellular ATP stimulation.

### ATP-mediated senescence in lung fibroblasts stimulates the growth of TNBC cells

**Figure 7:**
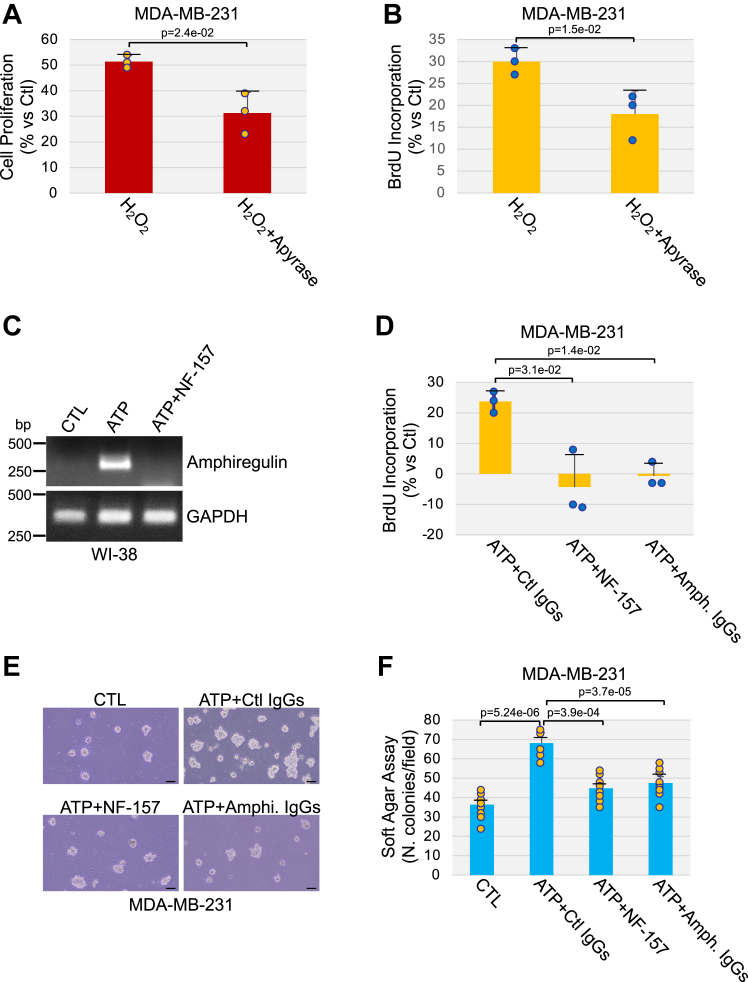
**P2Y11R-mediated release of amphiregulin by senescent fibroblasts promotes the growth and tumorigenic potential of TNBC cells.***A* and *B*, WI-38 fibroblasts were treated with sublethal oxidative stress (450 μM H_2_O_2_) for 2 h in the presence or absence of 4 U/ml apyrase. Cells were washed with PBS and recovered in complete medium for 10 days with or without 4 U/ml apyrase. Untreated cells were used as control. Conditioned medium was used to culture MDA-MB-231 breast cancer cells for 48 h. Cell proliferation was quantified by both cell counting (*A*) and BrdU incorporation assay (*B*). *C*, WI-38 fibroblasts were treated with 1.5 mM ATP for 10 days in the presence or absence of 40 μM NF-157. Untreated cells were used as control. The expression level of amphiregulin was quantified by RT-PCR analysis using amphiregulin-specific primers. GAPDH expression was quantified as control. *D*–*F*, WI-38 human diploid fibroblasts were treated with ATP (1.5 mM) for 10 days in the presence or absence of NF-157 (40 μM). Untreated cells served as control. Conditioned media was collected and conditioned medium from ATP-treated cells was incubated at 37 °C for 3 h with either a neutralizing amphiregulin Ab (4 μg/ml) or control IgGs (4 μg/ml). Conditioned media was then used to culture MDA-MB-231 breast cancer cells for either 2 days (*D*) or 10 days (*E* and *F*). In (*D*), MDA-MB-231 cell proliferation was quantified by BrdU incorporation assay. In (*E* and *F*), the tumorigenic potential of MDA-MB-231 cells was quantified by soft agar assay. Representative images are shown in (*E*), quantification is shown in (*F*). Values in *A*, *B*, *D*, and *F* represent means ± SD; statistical comparisons were made using the student’s *t* test. The scale bar represents 50 μm. BrdU, bromodeoxyuridine; H_2_O_2_, hydrogen peroxide; TNBC, triple-negative breast cancer.

Senescent cells can release factors that promote the growth of preneoplastic and neoplastic cells, including TNBC cells (18, 19, 34, 64). We asked whether oxidative stress-induced human senescent fibroblasts release factors that promote the growth of the TNBC cell line MDA-MB-231 in an ATP-dependent manner. To this end, we induced senescence in WI-38 fibroblasts by oxidative stress. We then used conditioned medium derived from senescent WI-38 cells to culture MDA-MB-231 cells. We find that the growth of MDA-MB-231 cells was enhanced when the cells were cultured in the presence of conditioned medium derived from senescent WI-38 fibroblasts following H_2_O_2_ stimulation, as compared to conditioned medium derived from control WI-38 cells, as shown by cell proliferation and BrdU incorporation assays (Fig. 7, *A* and *B*). The growth advantage of MDA-MB-231 cells elicited by conditioned medium derived from senescent fibroblasts was inhibited when WI-38 cells were subjected to oxidative stress in the presence of apyrase (Fig. 7, *A* and *B*). Interestingly, if breakdown of ATP with apyrase was carried out after the conditioned medium was removed from H_2_O_2_-treated and senescent WI-38 cells (and not during the 10-days culture period necessary to induce senescence), apyrase treatment failed to inhibit the growth enhancement of MDA-MB-231 cells (Fig. S6*A*). These data indicate that factors released by senescent fibroblasts in an extracellular ATP-dependent manner, but not ATP itself, are responsible for the growth stimulatory properties of senescent fibroblasts.

### Amphiregulin is a protumorigenic factor that is released by senescent fibroblasts upon P2Y11R stimulation

The epidermal growth factor receptor (EGFR) is overexpressed in several tumors of epithelial origin, including breast cancer (65, 66). Data suggest that EGFR plays an important role in TNBC (67, 68). TNBC cells lines, such as MDA-MB-231, show high expression levels of EGFR (69, 70, 71). Inhibition of EGFR signaling suppresses MDA-MB-231 cell proliferation (72). Amphiregulin is an autocrine growth factor that can bind and activate the EGFR (73). Amphiregulin is an EGFR ligand in TNBC cells. Loss of amphiregulin inhibits proliferation and invasion properties of breast cancer cells (74, 75, 76, 77).

Since we found that senescent cells secrete factors that stimulate the proliferation of TNBC cells in an ATP-dependent manner, we first asked whether amphiregulin was upregulated in lung fibroblasts that were induced to senesce by ATP stimulation. To this end, we treated WI-38 fibroblasts with ATP for 10 days to induce senescence. Amphiregulin mRNA expression was then determined by RT-PCR. We find that amphiregulin mRNA levels were increased in ATP-treated senescent WI-38 cells, as compared with nonsenescent cells (Fig. 7*C*). Interestingly, treatment with NF-157 prevented ATP-mediated amphiregulin upregulation (Fig. 7*C*). Thus, activation of P2Y11 receptor by ATP promotes amphiregulin expression in senescent WI-38 fibroblasts.

Then, to investigate the functional significance of P2Y11R-mediated upregulation of amphiregulin expression in senescent lung fibroblasts, we tested if ATP-dependent release of amphiregulin by senescent lung fibroblasts promoted proliferation of TNBC cells. More specifically, we cultured MDA-MB-231 breast cancer cells with conditioned medium derived from ATP-treated senescent WI-38 cells that were cultured in the presence of either NF-157 or a neutralizing antibody specific for amphiregulin. Conditioned medium from untreated and nonsenescent cells was used as control. The proliferation properties of MDA-MB-231 was measured by BrdU incorporation assay while the tumorigenic potential of these MDA-MB-231 cells was determined by soft agar assay. We show in Figure 7*D* that the growth enhancement of MDA-MB-231 cells induced by the conditioned medium derived from ATP-treated senescent WI-38 cells was inhibited if WI-38 cells were culture in the presence of the P2Y11 receptor antagonist or if their conditioned medium was incubated with an amphiregulin neutralizing antibody. Both NF-157 treatment and neutralizing amphiregulin immunoglobulin G (IgG) also inhibited the ability of conditioned medium derived from ATP-treated and senescent WI-38 fibroblasts to enhance the growth in soft agar of MDA-MB-231 cells (Fig. 7, *E* and *F*).

Similarly, conditioned medium derived from WI-38 cells that were induced to senesce by oxidative stress enhanced both the proliferation (Fig. S6*B*) and tumorigenic potential (Fig. S6, C and D) of MDA-MB-231 cells, which was inhibited by either P2Y11R inhibition with NF-157 in WI-38 fibroblasts or a neutralizing amphiregulin antibody. We conclude that amphiregulin is a protumorigenic factor that is released by senescent lung fibroblasts, following ATP stimulation, in a P2Y11R-dependent manner.

## Discussion

Although numerous cellular stressors can induce both premature senescence and the release of ATP in the extracellular space, the potential causal role of ATP-mediated signaling in cellular senescence remains largely unknown. In the present study, we describe for the first time that ATP-mediated purinergic stimulation mediated premature senescence induced by known senescence-inducing stressors, such as oxidative stress and UV-C light. Our findings are consistent with a study showing a correlation between irradiation-induced release of ATP and irradiation-induced accumulation of markers of senescence in glioblastoma cells (78). We also find that degradation of extracellular ATP inhibited but did not completely prevent oxidative stress-induced senescence. Since we find that ATP stimulation *per se* was sufficient to promote a premature senescent phenotype, we conclude that activation of purinergic signaling is sufficient to induce senescence and contributes to oxidative stress-induced senescence in human fibroblasts.

We find that the P2Y11 receptor was a key transducer of the purinergic signaling causally linking extracellular ATP to premature senescence. Importantly, our data show that P2Y2R was also expressed in human fibroblasts, and therefore we do not rule out the possibility that the P2Y2 purinergic receptor may contribute, together with P2Y11R, to the overall prosenescent P2Y-mediated signaling.

What is the signaling downstream of P2Y11R that drives ATP-mediated senescence? We describe that, upon ATP/P2Y11R-mediated and PLC-dependent calcium release from the ER, calcium entered mitochondria and drove oxidant generation. We find that excessive mitochondria-derived oxidative stress activated the p21 pathway and induced premature senescence. Interestingly, ATP stimulation increased MCU protein expression, which is indicative of the need by mitochondria to buffer elevated ER-derived cytoplasmic calcium and is consistent with mitochondrial calcium overload. Thus, our investigations indicate that a functional coupling between the ER and mitochondria drives cellular senescence in human fibroblasts and directly support a previous study showing that the forced contact between the ER and mitochondria promoted premature senescence (79). Moreover, our findings support previous data showing that loss of inositol 1,4,5-trisphosphate receptor, type 2 (ITPR2), which mediates calcium release from the ER, as well as loss of MCU inhibited oncogene-induced senescence and delayed replicative senescence (80). In addition, ITPR2 KO increased lifespan and limited age-associated phenotypes in mice (79). Interestingly, ablation of ITPR2 decreased the number of contacts between the mitochondria and the ER.

**Figure 8:**
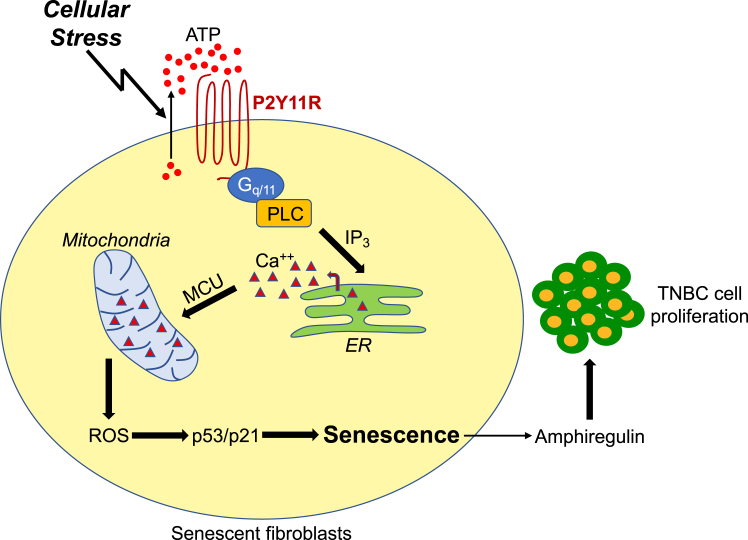
**Schematic diagram summarizing purinergic-dependent protumorigenic properties of senescent human fibroblasts.** Exogenous stress causes the release of ATP from human diploid fibroblasts. Extracellular ATP activates the P2Y11R receptor, which promotes the release of calcium from the endoplasmic reticulum in a G_q/11_/PLC/IP_3_-dependent manner. Calcium that is released from the ER accumulates in mitochondria through MCU. Mitochondrial calcium overload causes mitochondrial ROS generation. Increased ROS levels promote premature senescence through the activation of the p53/p21 pathway. Senescent fibroblasts release amphiregulin in an ATP/P2Y11R-dependent manner, which promotes the proliferation and tumorigenic potential of triple-negative breast cancer cells. ER, endoplasmic reticulum; IP_3_, inositol-1,4,5-trisphosphate; MCU, mitochondrial calcium uniporter; PLC, phospholipase C; ROS, reactive oxygen species.

Purinergic signaling modulates both cancer and immune cell behavior and, consequently, the host-tumor interaction and disease progression. The level of ATP is higher in the tumor microenvironment (TME), as compared to healthy tissues (81, 82, 83). Data show that ATP in the TME can stimulate cancer cell growth, survival, and metastatic potential by activating P2R-mediated signaling within the cancer cells (84, 85, 86, 87, 88). However, very little is known about ATP-mediated signaling within the fibroblastic cell component of the TME and its functional role as a regulator of tumor progression. We describe that conditioned medium derived from senescent fibroblasts, which were induced to senesce by ATP stimulation, enhanced the proliferation of MDA-MB-231 TNBC cells and their ability to grow in soft agar. Such enhancement was inhibited when either P2Y11R-mediated signaling was inhibited in the fibroblasts by a P2Y11R-specific antagonist or amphiregulin was inhibited by a specific antibody probe in the conditioned medium. Thus, our findings propose an additional level of protumorigenic regulation by purinergic signaling, in which a cell autonomous ATP-initiated and P2Y11R-mediated pathway in senescent fibroblasts induces the release of factors, such as amphiregulin, which enhance the tumorigenic potential of cancer cells (Fig. 8). Since TNBC is more likely than other types of breast cancer to metastasize to the lungs, and since WI-38 fibroblasts are of lung origin, we can speculate that senescent fibroblasts may create a permissible niche within the lungs, in a purinergic-dependent manner, which fuels TNBC cell growth. Moreover, our studies have a potential translational impact in the field of cancer: we envision prevention of premature senescence of fibroblasts within the TME, using either P2Y11R or PLC inhibitors, as possible alternative therapeutic options aimed at blocking or limiting the growth of TNBC cells at metastatic sites.

## Experimental procedures

### Materials

Antibodies were obtained from the following sources: anti-phospho-p53 (9284), and anti-γ-H2A.X (9718) IgGs were from Cell Signaling Technologies; anti-p21 (F-5), and anti-p53 (6243) IgGs were from Santa Cruz Biotechnology; anti-p16 ARC (mAb EP1551Y) was from Abcam. ATPγS, ARL 67156, KB-R7943 mesylate, U-73122, PPADS tetrasodium salt, 5-BDBD, A740003, NF-157, NF-546, BAPTA-AM, and CGS 15943 were from Tocris Bioscience. ATP, apyrase, caffeine, and quercetin were from Sigma-Aldrich. Silencer Select negative control siRNA and Silencer Select MCU siRNA were from Thermo Fisher Scientific. All other biochemical reagents were of the highest available purity and were commercially obtained.

### Cell culture

WI-38 human lung fibroblasts were cultured in Eagle’s minimal essential medium supplemented with 2 mM glutamine, 100 U/ml penicillin, 100 μg/ml streptomycin and 10% fetal bovine serum. MDA-MB-231 was cultured in Dulbecco’s modified Eagle's medium supplemented with 2 mM glutamine, 100 U/ml penicillin, 100 μg/ml streptomycin and 10% fetal bovine serum.

### Induction of premature senescence

#### H_2_O_2_-induced senescence

Cells were treated with sublethal doses of H_2_O_2_ (450 μM) for 2 h. Cells were washed twice with PBS and cultured in complete medium for the indicated periods of time.

#### UV-C-induced senescence

Cells were irradiated with a sublethal dose of UV-C light (10/m^2^). During irradiation, cells were deprived of growth medium. Cells were allowed to recover in complete medium for the indicated periods of time.

### Immunoblotting

Cells were collected in boiling sample buffer. Cellular proteins were resolved by SDS-PAGE (12.5% acrylamide) and transferred to Amersham Protran 0.2 μm nitrocellulose blotting membrane (GE HealthCare Life Sciences). Blots were incubated for 1 h and 15 min in tris buffered saline with tween-20 (TBST) (10 mM Tris–HCl, pH 8.0, 150 mM NaCl, and 0.2% Tween 20) containing 2% powdered skim milk and 1% bovine serum albumin. After three washes with TBST, membranes were incubated overnight with the primary antibody and washed three more times with TBST. Blots were then incubated for 1 h and 15 min with horseradish peroxidase-conjugated goat anti-rabbit/mouse IgG. Bound proteins were detected using an ECL detection kit (Pierce) according to the manufacturer’s protocol. Representative images are shown. Ponceau S staining is included to show equal total protein loading.

### Senescence-associated β-galactosidase activity assay

Senescence-associated β-galactosidase activity was measured using the Senescence β-Galactosidase Staining Kit according to the manufacturer’s protocol (Cell Signaling Technology). Average percent senescence was calculated from quantification of total cells and senescent cells in 10 fields of view per condition, using an inverted Olympus microscope (CKX53). Representative images of microscope fields are shown.

### Transfection of siRNA

siRNA was introduced into cells using Lipofectamine RNAiMax Reagent from Life Technologies according to the manufacturer’s protocol, using 40 pmol per well of 6-well culture plates.

### RNA isolation and RT-PCR

Cells were collected and total RNA was isolated using the RNeasy Mini kit from Qiagen. Equal amounts of RNA were treated with RNase-free DNase and subjected to reverse transcription using the Advantage RT-for-PCR kit from Clontech, according to the manufacturer’s protocol. PCR was then performed for each gene studied in its linear zone of amplification using the following primers: P2Y1R forward-agatctggacaactctcctct; P2Y1R reverse-ctcttggattgcaaatttgcc; P2Y2R forward-tctgcttcctgccattccacg; P2Y2R reverse-tcactgctgcccaacacatct; P2Y4R forward-tctgcacagtcgtcttctcgc; P2Y4R reverse-tatcctcaggcagggacacta; P2Y6R forward-actgtcatcggcttcctgctg; P2Y6R reverse-tgaagtagaagaggatggggt; P2Y11R forward-agcgttggtggccagtggtgt; P2Y11R reverse-cagtgctcttggcgtcctctg; P2Y12R forward-agaactgtaccggtcatacgt; P2Y12R reverse-tgttgcagaattggggcactt; P2Y13R forward-ggactgtttttatcctaatgc; P2Y13R reverse-ttctcccttgcatacatggta; P2Y14R forward-tcgtggccatcttctggattg; P2Y14R reverse-tccctaaacggctggcataga; P2X1R forward-tgtgacctggactggcacgta; P2X1R reverse-tcttctgcttgtagtagtgcc; P2X2R forward-gtccaccaggcccttacacac; P2X2R reverse-ctgctcagaaggggcagagat; P2X3R forward-cgtttctgagaaaagcagcgt; P2X3R reverse-ttcagcgtagtctcattcacc; P2X4R forward-catcatgggcatccaggtcaa; P2X4R reverse-aggactatgatgtcacacagc; P2X5R forward-gagtgccaccctcactattct; P2X5R reverse-cccagatgtgagctgctcaga; P2X6R forward-tggaatccgcttcgacatcct; P2X6R reverse-ctggtgtctgtgtctgactcc; P2X7R forward-gctgcttagaaaggaggcgac; P2X7R reverse-ggcaaagtcagccatgtcctg; Panx1 forward-cagaagacagatgttctcaaa; Panx1 reverse-ttcactgtctatgttcatacc; Panx2 forward-tacatcctcggcaccaagaag; Panx2 reverse-cctcgtacagcgtgttgatgt; Panx3 forward-atctgatcacatgcaggctga; Panx3 reverse-gtgtcaatattgtgcttctgg; Cx43 forward-tgccaaagactgtgggtctca; Cx43 reverse-ttgaaggtcgctggtccacaa; GAPDH forward-gcaaattccatggcaccgt; GAPDH reverse-tcgccccacttgattttgg.

### BrdU incorporation assay

Cell proliferation was measured and quantified using the Cell Proliferation ELISA, BrdU (colorimetric) kit (Roche). The kit was used according to the manufacturer’s protocol. Cells were incubated with BrdU labeling solution overnight at 37 °C. The absorbances were measured at 370 nm following substrate incubation using an ELISA plate reader.

### Cytosolic Ca^++^ measurements

Glass coverslips were loaded with Fura-2 AM (Thermo Fisher Scientific, F-1201) by incubating the cells in Fura-2 AM (1 μM) in Hanks′ balanced salt solution (HBSS; in mM: NaCl 138, KCl 5, KH_2_PO_4_ 0.03, Na_2_HPO_4_ 0.03, NaHCO_3_ 4, glucose 5.6, CaCl_2_ 2, MgCl_2_ 1, and Hepes 10, pH 7.4, 315 mOsmol/L) containing 0.2% (w/v) pluronic F-127 detergent for 20 min in the dark at room temperature. Following loading, the coverslips were placed into a flow chamber on an inverted microscope (IX73, Olympus; https://www.olympus-lifescience.com/) with a Hamamatsu digital camera (ORCA-Flash 4.0), an LED light system (pE-340fura, CoolLED) and a data analyzation computer running image analysis software (cellSens Dimension, Olympus). Cells were maintained in HBSS throughout the experiment by a gravity-fed perfusion system (World Precision Instruments). To record changes in [Ca^2+^]_i_, the cells were alternatively illuminated at 340 and 380 nm by UV light and imaged at 510 nm. Images were captured every 3 s and a 5-min baseline was collected before any manipulation. Agonists were applied to a static bath or through the perfusion system. Increases in intracellular calcium were detected as a change in the ratio of emission at each excitation wavelength (340/380). Data reported are the peak increase in Fura-2 ratio over baseline during application of agonist or area under the curve during a 3-min window before (baseline) and/or after agonist application.

### Mitochondrial Ca^++^ measurements

WI-38 cells were seeded onto 25 mm glass coverslips for 24 h in complete media. The coverslips were then mounted into an Attofluor cell chamber and incubated with 2 μM Rhod-2 AM and 100 nM Mito-Tracker Green in culture media for 30 min at room temperature. Cells were then washed 4x with HBSS, and in last wash, cells were left in HBSS for 10 min. The chambers were then mounted to a Leica DMi8 confocal microscope with a 20x objective. MitoTracker Green was excited with a 488 nm laser, and the corresponding emission was captured at 493 to 555 nm. Rhod-2 was excited with a 561 nm laser, and the corresponding emission was captured at 580 to 650 nm. Using Leica Applicate Suite X (https://www.leica-microsystems.com/) software, unbiased regions of interest were drawn around the mitochondria of cells. About 5 to 10 regions of interest were drawn for each coverslip. We normalized both Rhod-2 and mitotracker signals to t = 0 to account for any changes in focus, and then took the ratio of Rhod-2/Mitotracker signal to determine the magnitude of calcium entry. Maximum mitochondrial calcium entry was calculated as a fold change by dividing the maximum fluorescence signal by the average fluorescence of the baseline signal (from t = 0 to t = 60).

### Quantification of H_2_O_2_

Hydrogen peroxide was quantified using the Amplex Red Hydrogen Peroxide Assay kit (A22188) from Thermo Fisher Scientific, according to the manufacturer’s recommendations.

### Mitochondrial ROS measurement

Mitochondrial ROS production was measured by MitoSOX fluorescence in intact WI-38 cells. Cells were loaded with MitoSOX red (5 μM) and fluorescence (excitation 510 nm/emission 580 nm) was read for 30 min. The rate of production was normalized to protein content.

### Growth in soft agar

Cells (5 × 10^4^) were suspended in 3 ml of complete medium and 0.33% SeaPlaque low-melting temperature agarose. These cells were plated over a 2-ml layer of solidified complete medium and 0.5% agarose and allowed to settle to the interface between these layers at 37 °C. After 30 min, the plates were allowed to harden at room temperature for 30 min before returning to 37 °C. After 10 days, colonies were photographed under low magnification. The colonies in 60 randomly chosen fields from three independent plates were counted.

### ATP measurement

The level of extracellular ATP was quantified in the conditioned medium of WI-38 cells using the ATP bioluminescent assay kit from Sigma-Aldrich (cat. # FLASC), according to the manufacturer’s recommendations.

### Statistical analysis

Studies were performed at least in triplicates using three biological replicates to achieve statistically significant differences. The average ± SD is shown. Significance was calculated using the Student’s *t* test.

## Data availability

All data are contained within the manuscript.

## Supporting information

This article contains supporting information.

## Conflict of interest

The authors declare that they have no conflicts of interest with the contents of this article.
