# Supplementary material for: Purinergic signaling promotes premature senescence
Source: J Biol Chem. 2024 Mar 7;300(4):107145. doi: 10.1016/j.jbc.2024.107145 (PMC11002311; doi:10.1016/j.jbc.2024.107145)
Supplement: Supporting Information [file mmc1.pdf]

Supplemental Figure Legends

Figure S1. P2 purinergic receptor activation promotes stress-induced premature senescence. *(A,B)* WI-38 human diploid fibroblasts were treated with 450 µM H_2O_2 for 2 hours and recovered in complete medium for 10 days in the presence of either 4U/ml apyrase *(A)*, 500 µM PPADS *(B)*, or 5µM CGS 15943 *(B)*. Untreated cells served as control. Cellular senescence was assessed by senescence-associated β-galactosidase activity. Representative images are shown. *(C-E)* WI-38 fibroblasts were treated with sub-lethal UV-C light (10 J/m^2). Cells were recovered in complete medium for 10 days. Untreated cells served as control. Cells were stained to detect senescence-associated β-galactosidase activity. Representative images are shown in *(C)*, quantification is shown in *(D)*. *(E)* Cell lysates were subjected to immunoblotting analysis using antibodies specific for phospho-p53 and p21. Ponceau S staining shows equal total protein loading. (F) WI-38 cells were treated with sub-lethal UV-C light (10 J/m^2) and recovered in complete medium for 10 days. The level of extracellular ATP was quantified in the conditioned medium using an ATP bioluminescent assay kit. Values in D and F represent means ± St Dev; statistical comparisons were made using the student's t-test. Scale bar = 50 µm.

Figure S2. Treatment with apyrase inhibits ATP-induced premature senescence. *(A-C)* Human diploid WI-38 fibroblasts were treated with 1.5 mM ATP for 10 days in the presence or absence of 4U/ml apyrase. Untreated cells were used as control. *(A,B)* Cells were subjected to senescence-associated β-galactosidase activity staining. Representative images are shown in *(A)*, quantification is shown in *(B)*. *(C)* Cells were collected and cell lysates were subjected to immunoblot analysis using an antibody probe specific for p21. Ponceau S staining shows equal total protein loading. *(D)* WI-38 cells were treated with 1.5 mM adenosine for 10 days. Senescence was quantified by senescence-associated β-galactosidase activity. Values in B and D represent means ± St Dev; statistical comparisons were made using the student's t-test. Scale bar = 50 µm.

Figure S3. Both ATP-induced and oxidative stress-induced premature senescence are inhibited by a P2Y11R antagonist. Treatment with a P2Y11R agonist is sufficient to promotes premature senescence. *(A,B)* WI-38 cells were treated with 400 µM ARL 67156 for 10 days in the presence of either 40 µM NF-157 or 40 µM 5-BDBD. Untreated cells were used as control. Cells were stained to detect senescence-associated β-galactosidase activity. Representative images are shown in *(A)*, quantification is shown in *(B)*. *(C)* WI-38 fibroblasts were treated with 1.5 mM ATP in the presence or absence of 40µM NF-157. Untreated cells served as control. Cell proliferation was quantified by BrdU incorporation assay. *(D,E)* WI-38 fibroblasts were treated with 450 µM H_2O_2 for 2 hours and recovered in complete medium for 10 days in the presence or absence of 40 µM NF-157. Untreated cells served as control. Cellular senescence was assessed by senescence-associated β-galactosidase activity. Representative images are shown in *(D)*, quantification is shown in *(E)*. *(F)* WI-38 cells were stimulated with NF-546 (80 µM) for 10 days. Untreated cells served as control. Cell proliferation was quantified by BrdU incorporation assay. Values in B, C, E, and F represent means ± St Dev; statistical comparisons were made using the student's t-test. Scale bar = 50 µm.

Figure S4. Intracellular calcium level is not increased in ATP-treated cells once the cells develop a senescent phenotype. Intracellular calcium was quantified in senescent WI-38 cells

loaded with Fura-2 AM, 10 days after treatment with 1.5 mM ATP. Untreated cells served as control. Values represent means ± St Dev; statistical comparisons were made using the student's t-test.

Figure S5. Inhibition of MCU impairs ATP-induced upregulation of p21. Quercetin inhibits ATP-induced senescence. *(A)* WI-38 human fibroblasts were stimulated for 10 days with ATP (1.5 mM) in the presence or absence of different concentrations of Ru-360 (3 μM, 9 μM, and 27 μM). Untreated WI-38 cells were used as control. Cells lysates were subjected to immunoblotting analysis using a p21-specific antibody probe. Ponceau S staining shows equal total protein loading. *(B-D)* WI-38 cells were treated with 1.5 mM ATP for 10 days in the presence or absence of quercetin (100μM). Untreated cells were used as control. *(B,C)* Cells were stained to detect senescence-associated β-galactosidase activity. Representative images are shown in *(B)*, quantification is shown in *(C)*. *(D)* Cells were collected and cell lysates were subjected to immunoblot analysis using an antibody probe specific for the senescence marker p21. Ponceau S staining shows equal total protein loading. Values in C represent means ± St Dev; statistical comparisons were made using the student's t-test. Scale bar = 50 μm.

Figure S6. P2Y11R-dependent release of amphiregulin by oxidative stress-induced senescent fibroblasts promotes proliferation and the tumorigenic potential of TNBC cells. *(A)* WI-38 fibroblasts were treated with sublethal oxidative stress (450 μM H_2O_2) for 2 hours. Cells were washed with PBS and recovered in complete medium for 10 days. Untreated cells were used as control. Conditioned medium was collected, incubated for 3 hours in the presence or absence of 4U/ml apyrase (apyrase*), and used to culture MDA-MB-231 breast cancer cells for 48 hours. Cell proliferation was quantified by BrdU incorporation assay. *(B-D)* WI-38 human diploid fibroblasts were treated with H_2O_2 (450 μM) for 2 hours and recovered in complete medium for 10 days in the presence or absence of NF-157 (40 μM). Untreated cells served as control. Conditioned media was collected and conditioned medium from H_2O_2-treated cells was incubated at 37 ^{o}C for 3 hours with either a neutralizing amphiregulin Ab (4μg/ml) or control IgGs (4μg/ml). Conditioned media was then used to culture MDA-MB-231 breast cancer cells for either 2 days *(B)* or 10 days *(C,D)*. In *(B)*, MDA-MB-231 cell proliferation was quantified by BrdU incorporation assay. In *(C,D)*, the tumorigenic potential of MDA-MB-231 cells was quantified by soft agar assay. Representative images are shown in *(C)*, quantification is shown in *(D)*. Values in A, B, and D represent means ± St Dev; statistical comparisons were made using the student's t-test. Scale bar = 50 μm.

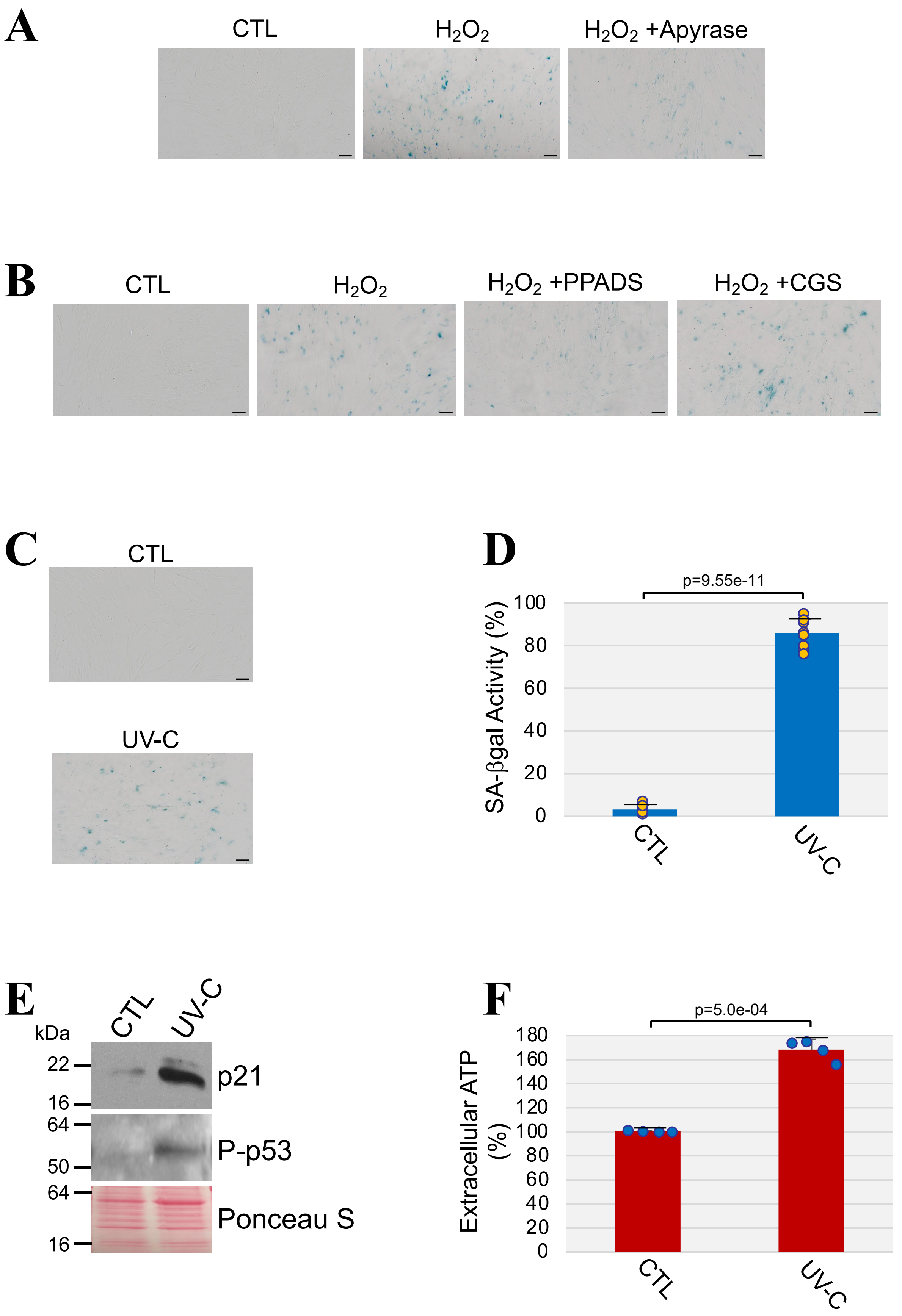
A
CTL
H_2O_2
H_2O_2 +Apyrase
B
CTL
H_2O_2
H_2O_2 +PPADS
H_2O_2 +CGS
C
CTL
UV-C
D
p=9.55e-11
SA-βgal Activity (%)
100
80
60
40
20
0
CTL
UV-C
E
CTL
UV-C
kDa
22
16
64
50
64
16
p21
P-p53
Ponceau S
F
p=5.0e-04
Extracellular ATP (%)
180
160
140
120
100
80
60
40
20
0
CTL
UV-C

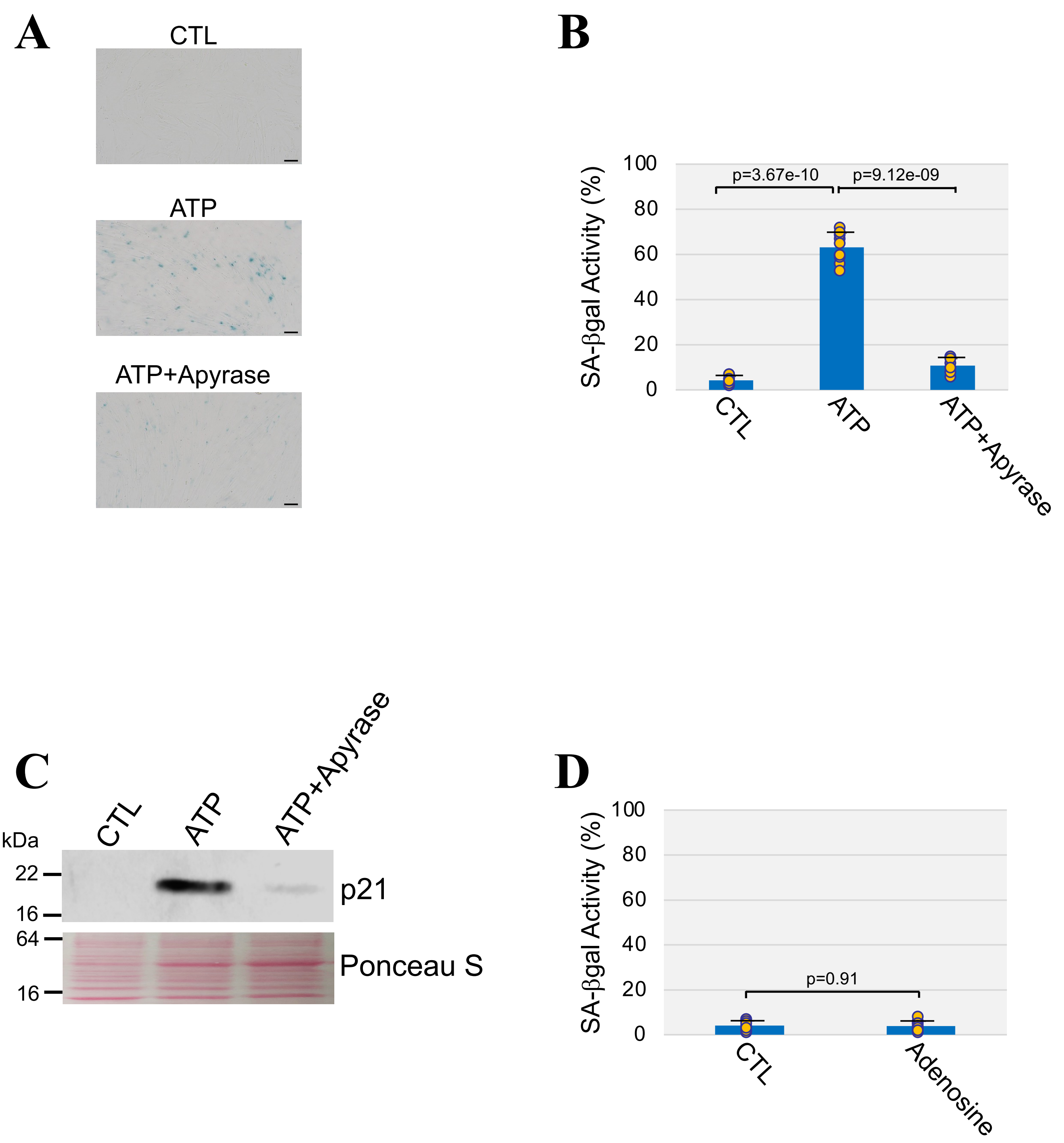
A
CTL
ATP
ATP+Apyrase
B
SA-βgal Activity (%)
100
80
60
40
20
0
p=3.67e-10
p=9.12e-09
CTL
ATP
ATP+Apyrase
C
CTL
ATP
ATP+Apyrase
kDa
22
16
64
16
p21
Ponceau S
D
SA-βgal Activity (%)
100
80
60
40
20
0
p=0.91
CTL
Adenosine

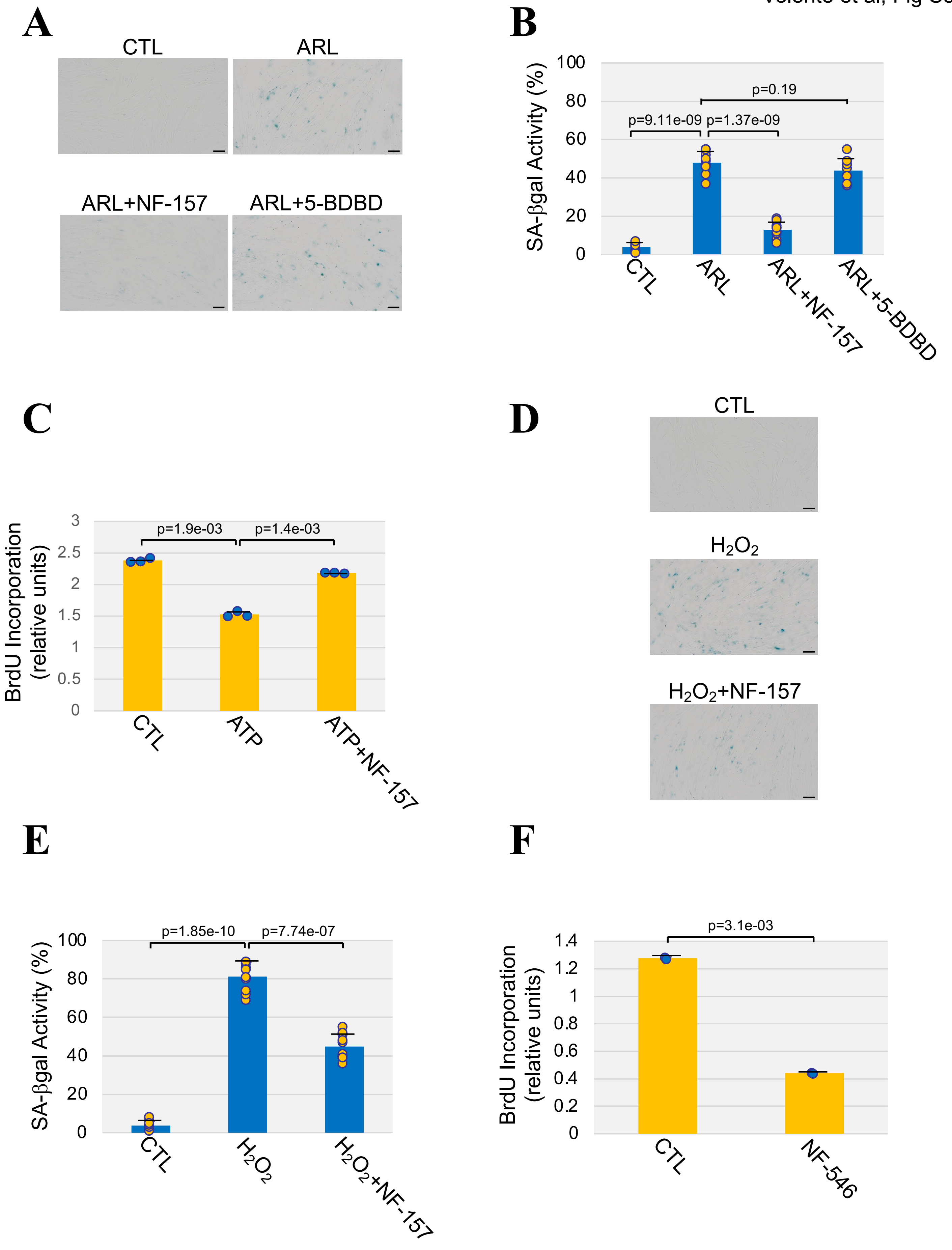
A
CTL
ARL
ARL+NF-157
ARL+5-BDBD
B
SA-βgal Activity (%)
100
80
60
40
20
0
p=0.19
p=9.11e-09
p=1.37e-09
CTL
ARL
ARL+NF-157
ARL+5-BDBD
C
BrdU Incorporation (relative units)
3
2.5
2
1.5
1
0.5
0
p=1.9e-03
p=1.4e-03
CTL
ATP
ATP+NF-157
D
CTL
H2O2
H2O2+NF-157
E
SA-βgal Activity (%)
100
80
60
40
20
0
p=1.85e-10
p=7.74e-07
CTL
H2O2
H2O2+NF-157
F
BrdU Incorporation (relative units)
1.4
1.2
1
0.8
0.6
0.4
0.2
0
p=3.1e-03
CTL
NF-546

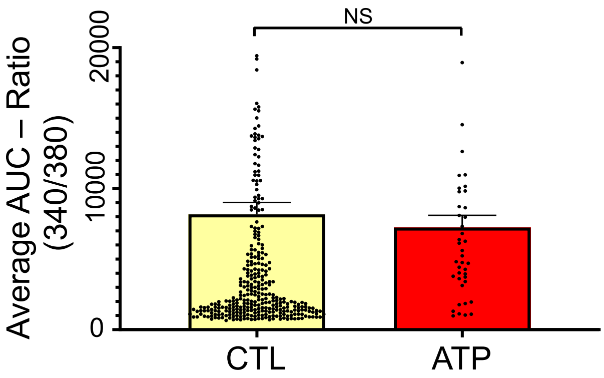
NS
Average AUC – Ratio (340/380)
20000
10000
0
CTL
ATP

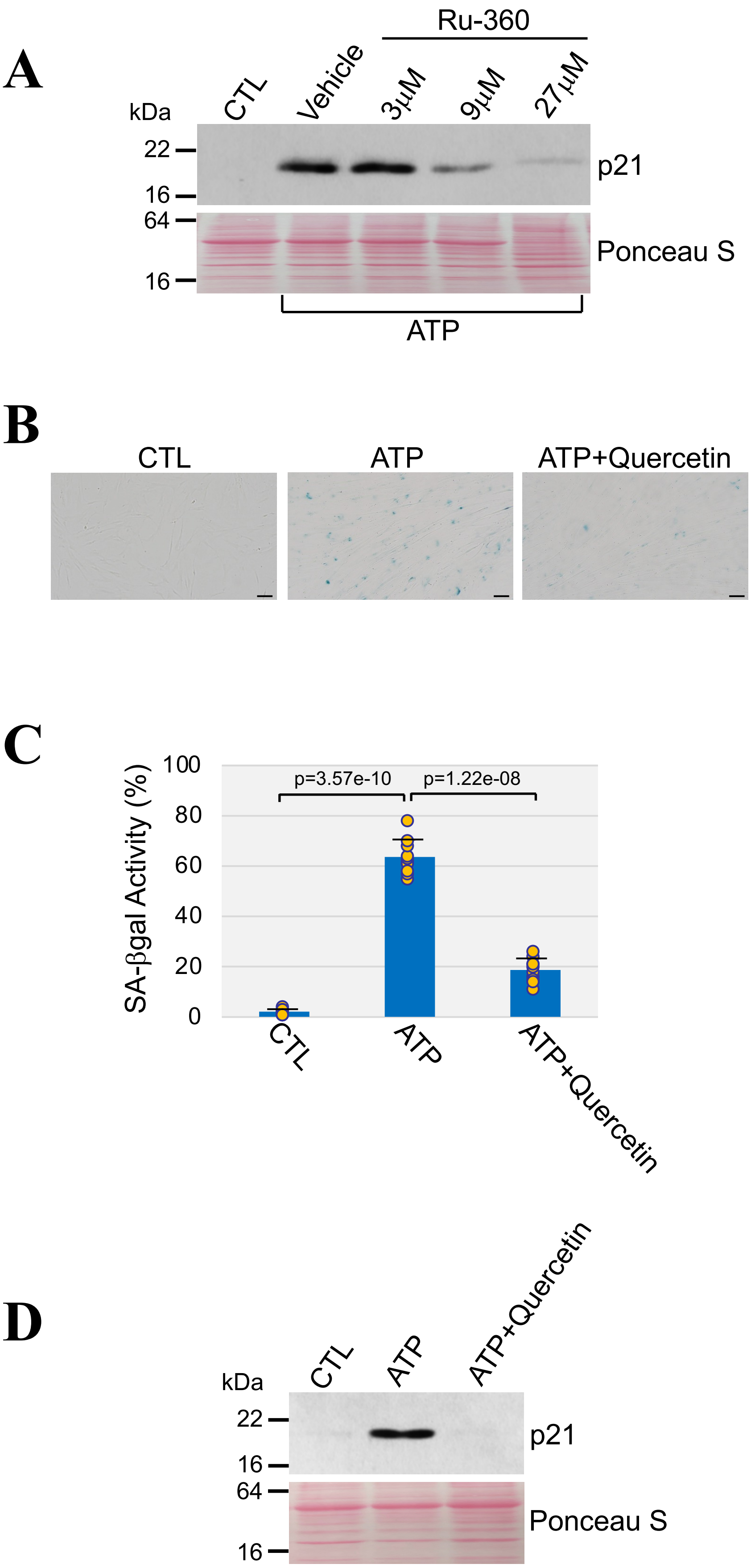
A
Ru-360
kDa
CTL
Vehicle
3μM
9μM
27μM
22
16
64
16
p21
Ponceau S
ATP
B
CTL
ATP
ATP+Quercetin
C
SA-βgal Activity (%)
100
80
60
40
20
0
p=3.57e-10
p=1.22e-08
CTL
ATP
ATP+Quercetin
D
kDa
CTL
ATP
ATP+Quercetin
22
16
64
16
p21
Ponceau S

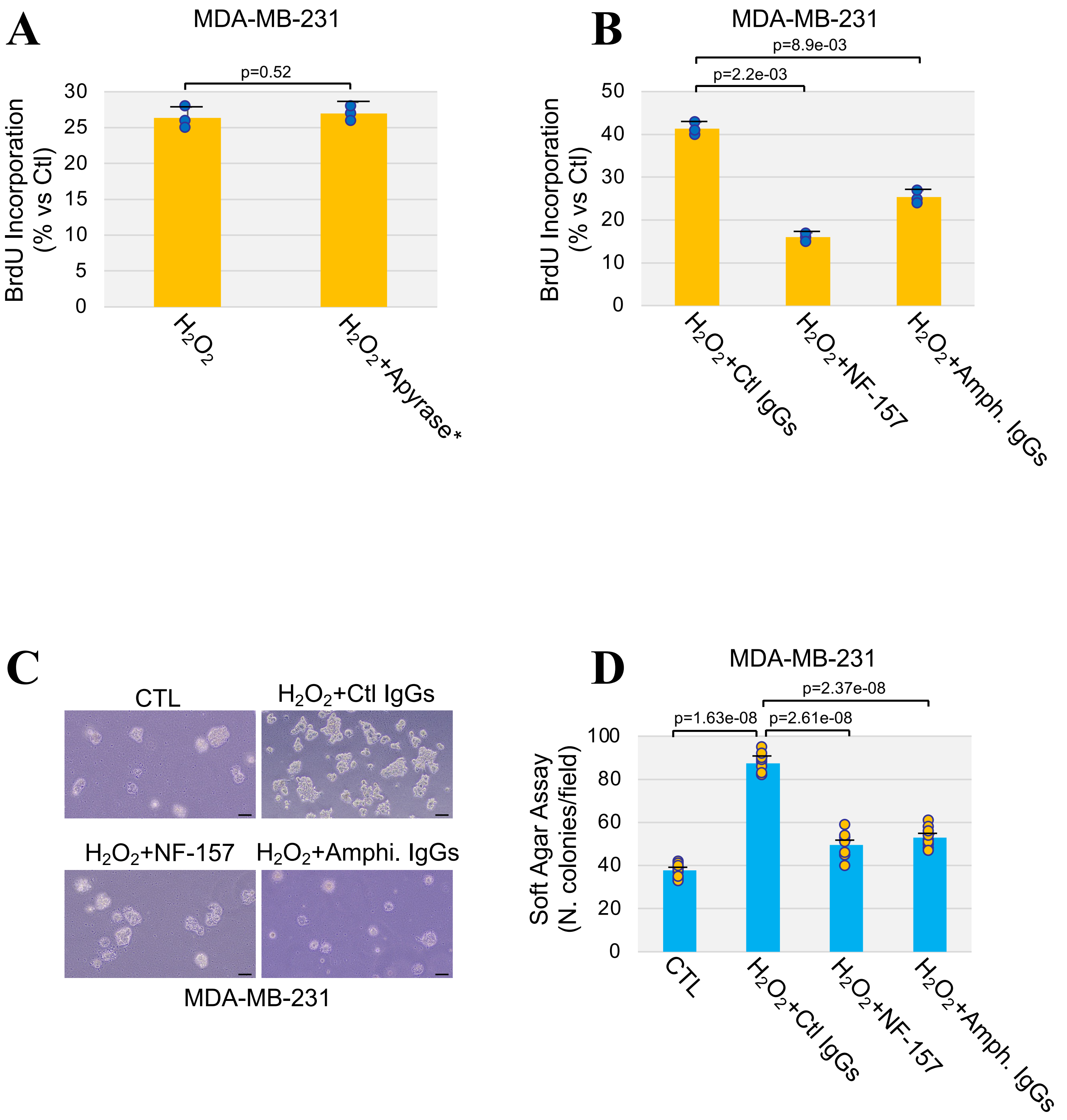
A
MDA-MB-231
p=0.52
BrdU Incorporation
(% vs Ctl)
30
25
20
15
10
5
0
H_2O_2
H_2O_2+Apyrase*
B
MDA-MB-231
p=8.9e-03
p=2.2e-03
BrdU Incorporation
(% vs Ctl)
50
40
30
20
10
0
H_2O_2+Ctl IgGs
H_2O_2+NF-157
H_2O_2+Amph. IgGs
C
CTL
H_2O_2+Ctl IgGs
H_2O_2+NF-157
H_2O_2+Amphi. IgGs
MDA-MB-231
D
MDA-MB-231
p=2.37e-08
p=1.63e-08
p=2.61e-08
Soft Agar Assay
(N. colonies/field)
100
80
60
40
20
0
CTL
H_2O_2+Ctl IgGs
H_2O_2+NF-157
H_2O_2+Amph. IgGs
